# Study on coal and gas outburst prediction technology based on multi-model fusion

**DOI:** 10.3389/fdata.2025.1623883

**Published:** 2025-10-20

**Authors:** Qian Xie, Junsheng Yan, Zhenhua Dai, Wengang Du, Xuefei Wu

**Affiliations:** ^1^CCTEG Xi‘an Transparent Geology Technology Co., Ltd., Xi'an, China; ^2^National Key Laboratory of Intelligent Coal Mining and Rock Stratum Control, Beijing, China; ^3^CCTEG Xi'an Research Institute (Group) Co., Ltd., Xi'an, China; ^4^CCTEG China Coal Research Institute, Beijing, China

**Keywords:** artificial intelligence, coal and gas outbursts prediction, multi-model fusion, XGBoost, attention mechanism

## Abstract

The rapid advancement of artificial intelligence (AI) and machine learning (ML) technologies has opened up novel avenues for predicting coal and gas outbursts in coal mines. This study proposes a novel prediction framework that integrates advanced AI methodologies through a multi-model fusion strategy based on ensemble learning and model Stacking. The proposed model leverages the diverse data interpretation capabilities and distinct training mechanisms of various algorithms, thereby capitalizing on the complementary strengths of each constituent learner. Specifically, a Stacking-based ensemble model is constructed, incorporating Support Vector Machines (SVM), Random Forests (RF), and k-Nearest Neighbors (KNN) as base learners. An attention mechanism is then employed to adaptively weight the outputs of these base learners, thereby harnessing their complementary strengths. The meta-learner, primarily built upon the XGBoost algorithm, integrates these weighted outputs to generate the final prediction. The model's performance is rigorously evaluated using real-world coal and gas outburst data collected from a mine in Pingdingshan, China, with evaluation metrics including the F1-score and other standard classification indicators. The results reveal that individual models, such as XGBoost, SVM, and RF, can effectively quantify the contribution of input feature importance using their inherent mechanisms. Furthermore, the ensemble model significantly outperforms single-model approaches, particularly when the base learners are both strong and mutually uncorrelated. The proposed ensemble framework achieves a markedly higher F1-score, demonstrating its robustness and effectiveness in the complex task of coal and gas outburst prediction.

## 1 Introduction

Coal and gas outbursts remain a significant threat to the safety of coal mine workers in China. In geologically complex regions, the deepening of mining activities further aggravates subsurface conditions, increasing both the frequency and severity of outburst-related incidents (Ou et al., 2023; Fu et al., 2022). Therefore, the development of accurate and reliable predictive models for coal and gas outbursts is critically important.

Extensive research has been conducted on predictive models for coal and gas outbursts, resulting in the development of a variety of approaches. These include the initial velocity method for borehole outbursts (Wang et al., 2020a), the drilling cuttings index method (Wang et al., 2020b), mathematical evaluation models (Soleimani et al., 2023; Hassan et al., 2017; Zhou et al., 2019; Rudakov and Sobolev, 2019; Yang et al., 2023), and AI-based models (Qiao et al., 2019; Anani et al., 2024; Li et al., 2024; Zhu et al., 2023; Song et al., 2021; Wang et al., 2023), all of which have demonstrated varying degrees of effectiveness. The rapid progress in AI technology has provided new opportunities for enhancing prediction accuracy. For instance, Fan et al. improved the SVM model using the firefly algorithm (FA) to predict coal and gas outbursts and validated its overall performance (Fan et al., 2023). Liu et al. used a least squares SVM optimized with the particle swarm optimization (PSO) algorithm, confirming its effectiveness using gas outburst data from the Jiulishan Coal Mine in Jiaozuo City, China (Liu et al., 2021). Furthermore, Zheng et al. (2023) used XGBoost to predict and analyze the contribution rate distribution of coal and gas outburst indicators. However, these aforementioned studies tend to treat coal and gas outburst prediction as an isolated task. Given the inherent uncertainties and complex underlying mechanisms of such predictions, multiple hypotheses may perform well on the training set. Relying on a single model may suffer from poor generalization due to its susceptibility to randomness and overfitting. To address these limitations, we proposed a novel multi-model fusion prediction method that integrates an attention mechanism for analyzing the contribution rates of coal and gas data (Zhao et al., 2024a; Lin et al., 2020). Initially, Pearson's correlation analysis was conducted to identify and select strongly correlated features as model inputs. Subsequently, within the Stacking ensemble framework, a coal and gas outburst prediction model that integrates multiple learners was constructed to capture a more comprehensive data observation space. Finally, the efficacy of the proposed model was rigorously validated using real-world data from the Pingdingshan Coal Mine in China. The results unequivocally demonstrate that the Stacking-based ensemble method with multi-model fusion achieves robust predictive performance for coal and gas outburst events.

## 2 Data analysis

### 2.1 Research overview and data sources

**Figure 1:**
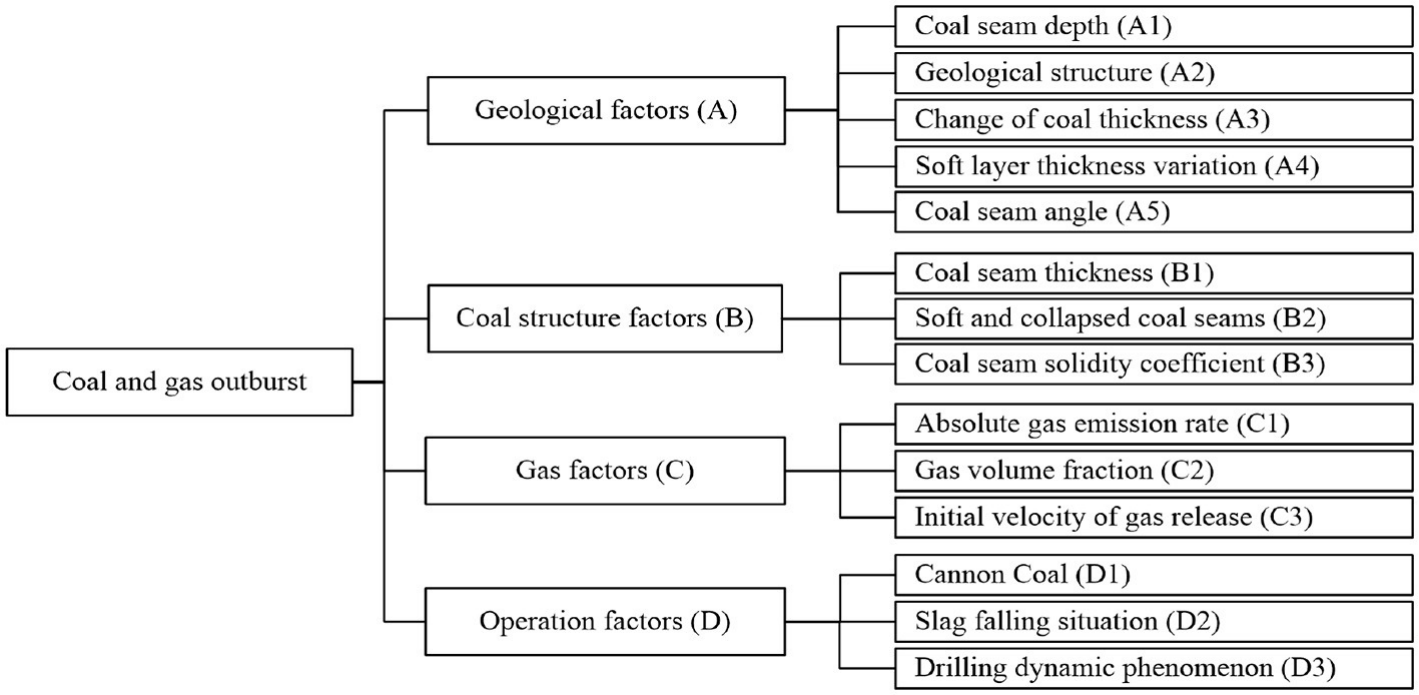
Factors influencing coal and gas outbursts.

Coal and gas outbursts are influenced by four main factors: geological conditions, coal seam characteristics, gas-related factors, and operational practices. Based on field observations, these factors are further subdivided into 14 specific elements (He et al., 2010) (Figure 1). The risk level (*L*) of coal and gas outbursts is classified into five categories based on the amount of ejected coal (Table 1).

**Table 1:** Coal and gas outburst hazard levels.

**Risk level**	**Outburst category**	**Coal thrown quantity (*Q*/*t*)**
I	No outburst	0
II	Small outburst	0 < *Q* ≤ 50
III	Medium outburst	50 < *Q* ≤ 100
IV	Large outburst	100 < *Q* ≤ 500
V	Extra-large outburst	500 < *Q*

**Figure 2:**
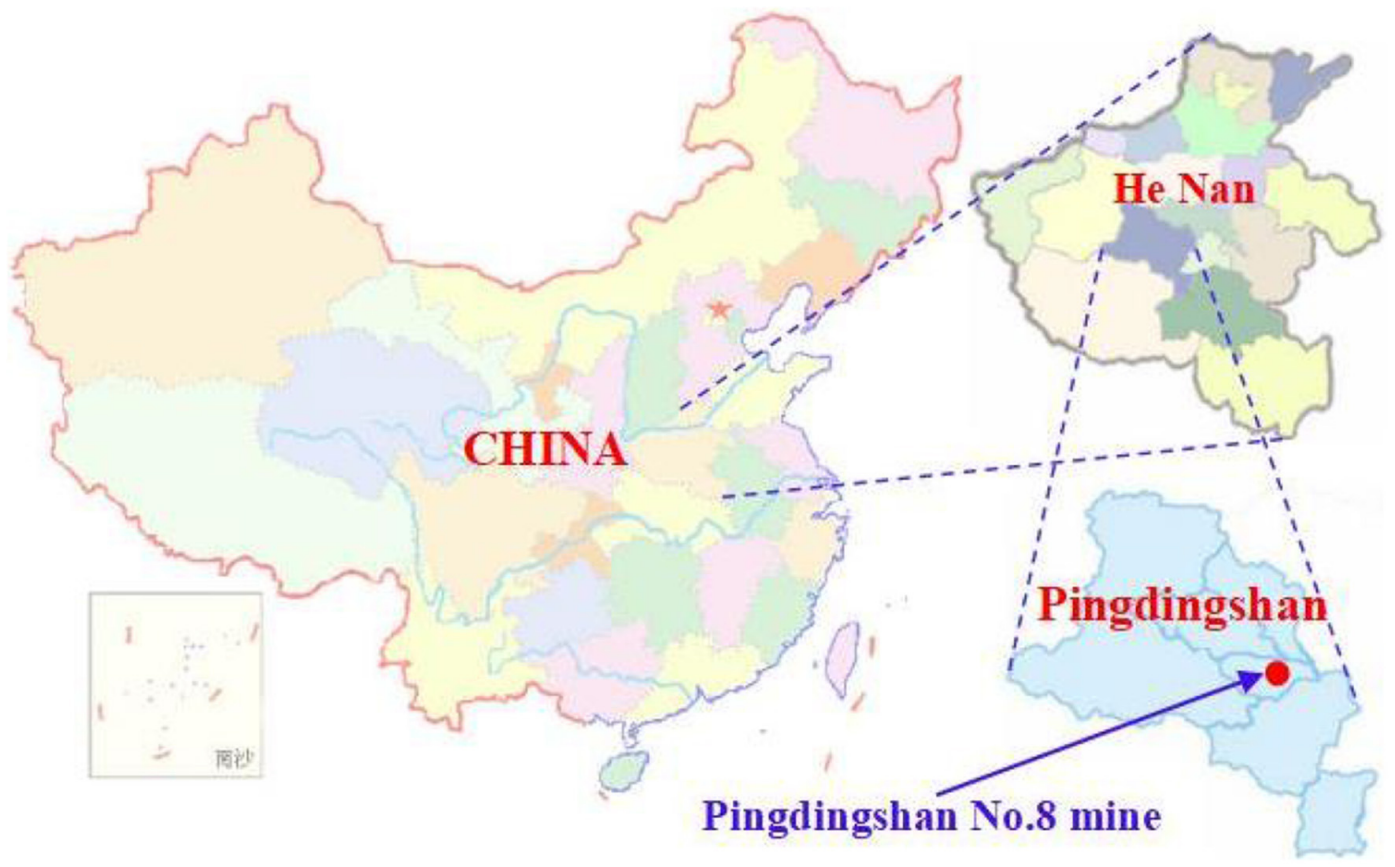
Geographical location of the No. 8 mine in Pingdingshan, China.

In this study, we used coal and gas outburst data collected from a coal mine in Pingdingshan over a period spanning from 1984 to 2009 (Xie et al., 2018). The first 50 data points were selected as the training set, while the final 10 data points were reserved for testing purposes. The coal and gas outburst data from the Pingdingshan Coal Mine are summarized in Table 2. The geographical location of Pingdingshan is shown in Figure 2.

**Table 2:** Coal and gas outburst data from a coal mine in Pingdingshan.

**Number**	**A1**	**A2**	**A3**	**A4**	**A5**	**B1**	**B2**	**B3**	**C1**	**C2**	**C3**	**D1**	**D2**	**D3**	***Q*/*t***	***L***
1	535	3	3	1	1	5.4	1	0.32	0.66	0.4	11.23	3	1	1	19.7	II
2	522	3	1	3	1	4.8	1	0.35	1.93	0.5	10.17	1	3	1	16	II
3	584	3	1	1	5	3.2	3	0.2	2.38	0.53	12.06	3	1	5	132	IV
4	484	3	3	1	3	4.81	1	0.53	5.25	0.5	4.75	1	1	1	12	II
5	566	5	1	1	1	3.5	1	0.51	0.36	0.2	9	1	1	1	30	II
6	463	1	1	3	1	4.81	3	0.26	6.24	0.6	4.8	3	3	1	46	II
7	490	3	1	1	1	4.81	1	0.49	6.24	0.6	7.81	3	1	1	28	II
8	424	1	1	1	1	3.65	3	0.51	0.47	0.27	8.53	3	1	1	6	II
9	535	3	1	1	1	5.2	3	0.29	3.68	0.49	17.14	3	1	1	62	III
10	566	5	3	3	3	3.5	1	0.38	1.04	0.75	8.57	3	3	3	144.6	IV
11	563.4	5	1	1	1	3.7	1	0.57	0.76	0.4	8.76	1	1	3	53	II
12	564	5	1	1	1	3.7	1	0.57	0.7	0.38	7.24	1	1	1	0	I
13	485	5	3	5	3	3.3	1	0.11	7.8	1.2	19.65	3	3	5	450	IV
14	482	3	1	1	1	3.4	1	0.14	7.6	1.3	18.64	1	1	1	0	I
15	623	3	3	1	3	4.5	1	0.35	0.44	0.3	14.27	3	1	1	22	II
16	584	3	1	1	3	3.2	1	0.24	2.32	0.5	11.86	1	1	3	0	I
17	557.6	1	1	1	1	3.1	3	0.54	0.52	0.4	11.38	3	3	1	43	II
18	557.6	1	3	1	1	3.4	3	0.15	0.78	0.6	18.85	3	3	3	240	IV
19	557.6	1	1	1	1	3.2	3	0.46	0.36	0.5	9.48	1	1	1	0	I
20	486	1	1	1	1	3.5	3	0.29	0.33	0.38	12.56	3	1	1	22	II
21	529.8	3	1	3	1	4.3	3	0.67	0.42	0.32	11.48	3	3	1	5	II
22	583	1	1	1	1	4.5	3	0.43	1.15	0.7	9.18	3	3	3	10	II
23	583	1	1	1	1	4.7	1	0.46	1.05	0.66	9.24	1	1	1	0	I
24	533	5	3	3	3	4.1	3	0.23	0.34	0.2	12.57	5	3	3	440	IV
25	530	5	1	1	1	4.1	1	0.36	0.32	0.18	12.38	1	3	1	0	I
26	622	3	1	1	1	3	1	0.32	2.31	0.5	20.19	3	1	1	64	III
27	573	1	1	1	1	4.1	3	0.5	0.79	0.34	7.33	3	1	1	16	II
28	537.9	3	1	1	3	5.3	1	0.19	0.22	0.8	23.91	3	3	3	138	IV
29	562	3	1	1	1	5.25	1	0.47	4.5	0.5	14.28	1	3	1	12.5	II
30	540	1	1	1	1	4.8	1	0.31	0.62	0.32	12.33	1	1	1	0	I
31	540	1	1	3	1	4.8	3	0.27	0.52	0.3	12.05	1	1	1	8	II
32	457	1	1	1	3	3.5	3	0.15	1.95	0.6	5.09	1	5	1	478	IV
33	460	1	1	1	1	3.5	1	0.38	1.38	0.52	4.68	1	1	1	0	I
34	589	3	1	1	1	3.2	3	0.24	2.1	0.6	11.05	1	1	1	4.6	II
35	636.4	5	3	3	5	3.2	3	0.15	3.08	0.46	18.25	3	5	5	396	IV
36	584	3	1	1	5	3.2	3	0.25	2.94	0.7	14.18	5	3	5	215	IV
37	564.6	5	1	1	1	3.5	1	0.48	0.78	0.6	9.27	3	1	1	44	II
38	480	1	1	1	1	4.81	1	0.53	5.25	0.5	4.75	1	1	1	0	I
39	840	7	3	3	5	4.5	3	0.17	1.15	0.25	23.52	3	3	5	551	IV
40	838	5	1	1	1	4.5	1	0.26	1.03	0.25	20.86	1	1	3	0	I
41	566	5	1	3	1	3.5	1	0.51	0.48	0.6	7.93	1	1	1	55	III
42	620	1	1	1	1	3	1	0.34	1.83	0.46	18.75	1	1	1	0	I
43	800	5	1	3	1	3.3	3	0.18	0.42	0.22	15.89	1	3	5	190	IV
44	820	5	1	1	1	4.5	1	0.21	1.22	0.28	20.31	1	1	1	0	I
45	614	1	1	1	1	4.5	1	0.55	5.4	0.3	9.87	3	1	1	7	II
46	697	1	1	1	1	4.1	1	0.35	0.58	0.12	15.91	3	1	1	14	II
47	629	3	1	1	1	4.5	1	0.34	0.99	0.18	15.67	1	1	3	32	II
48	490	3	1	1	1	3.2	1	0.51	0.85	0.15	14.32	1	5	1	34	II
49	652	1	1	1	1	4	1	0.54	0.5	0.52	12.03	3	1	3	5	II
50	820	7	1	1	1	4.5	1	0.19	1.22	0.3	24.71	5	1	1	115	IV
51	554	1	3	3	1	5.4	1	0.28	0.35	0.3	15.47	3	1	1	27	II
52	482	3	3	1	3	4.81	1	0.53	6.24	0.6	4.75	1	1	1	20	II
53	550	1	1	1	1	5.4	1	0.4	0.28	0.25	10.48	1	1	1	0	I
54	606	1	3	1	1	2	1	0.41	0.34	0.45	7.99	3	1	1	16	II
55	557.6	3	3	1	3	3	3	0.24	1.5	0.5	21.06	3	1	3	180	IV
56	563	1	1	1	1	3.5	1	0.55	0.42	0.35	6.21	1	1	1	0	I
57	487	3	3	1	3	4.81	1	0.53	5.25	0.5	4.75	1	1	1	10	II
58	583	1	3	1	1	4.8	1	0.34	1.24	0.75	10.34	3	3	1	20	II
59	580	1	1	1	1	3.4	1	0.26	2.7	0.52	12.27	1	3	1	0	I
60	520	3	1	3	1	4.5	1	0.26	1.38	0.4	12.74	1	1	3	45.5	II

### 2.2 Data process

Before feeding the training data into the model, it is crucial to carefully preprocess the dataset by identifying and addressing any anomalies.

Step 1: The Pauta criterion was applied to establish the outlier threshold. Data points that deviated from the mean by more than three standard deviations were considered outliers. These outliers were then removed, and their corresponding entries were set to null values.

Step 2: Missing values were then filled by extracting the five data points preceding and following each missing entry. In this study, Lagrange interpolation was used to estimate the missing data, as shown in Equations 1, 2.


(1) $$\begin{array}{l} L_{n}\left(x\right)=\sum_{i=o}^{n}l_{i}\left(x\right)y_{i} \end{array}$$

(2) $$\underset{\;j\neq i}{l_{i}(x)=\prod_{j=0}^{n}\frac{x-x_{j}}{x_{i}-x_{j}},}$$

where *l*_*i*_(*x*) represents the interpolation basis function; *L*_*n*_(*x*) denotes the interpolated value of the missing data; *y*_*i*_ is a known (non-missing) value; *x* is the index corresponding to the missing value; *x*_*i*_ denotes the index of the known data point; and *x*_*j*_ is the interpolation node.

### 2.3 Correlation analysis

**Figure 3:**
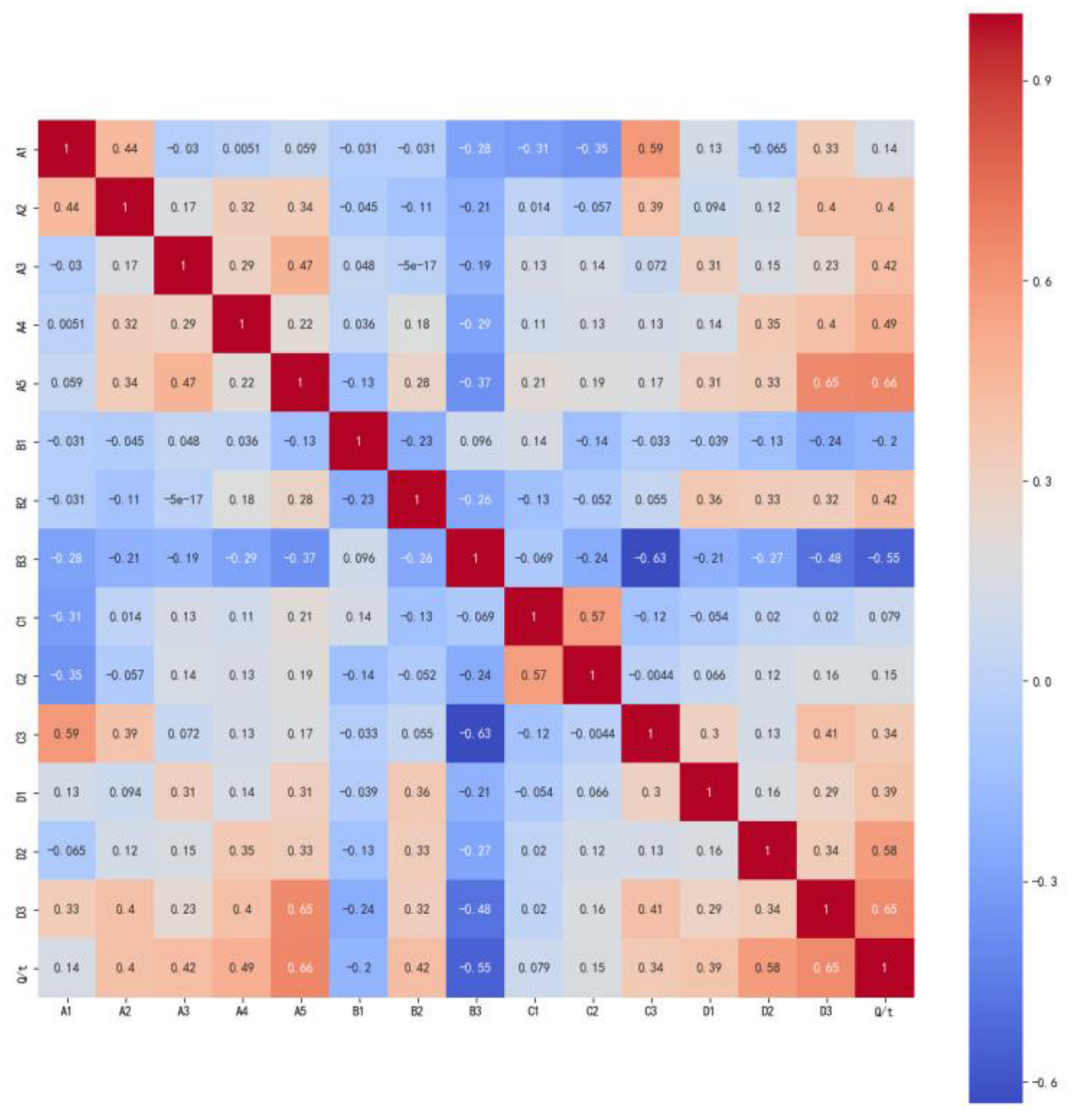
Pearson's correlation analysis of factors affecting coal and gas outbursts.

The primary indicator for determining the severity of a coal and gas outburst was the quantity of coal thrown (Table 1). As shown in Figure 3, Pearson's correlation analysis was performed to rigorously investigate both the interrelationships among the various influencing factors and their individual correlations with the quantity of coal thrown, as defined by Equation 3.


(3) $$\begin{array}{l} \rho_{X,Y}=\frac{cov\left(X,Y\right)}{\sigma_{X}\sigma_{Y}}, \end{array}$$

where *cov*(*X, Y*) is covariance and σ_*X*_ *and σ*_*Y*_ are the standard deviations of *X* and *Y*, respectively.

Following the strong and weak correlation partitions established by Zhang et al. (2022), the correlation results presented in Figure 3 were subsequently classified. The outcomes of this classification are comprehensively detailed in Table 3. As demonstrated in Table 3, this study meticulously selected six factors exhibiting medium to high correlation levels as input variables for the model, specifically including A5, B3, D2, A4, B2, and A3.

**Table 3:** Pearson's correlation analysis results.

**Degree of correlation**	**Value range**	**Results**
Strong correlation	(0.6,0.8]	A5 and B3
Moderately correlation	(0.4,0.6]	D2, A4, B2, and A3
Weak correlation	(0/2,0.4]	A2, D1, and C3
No correlation	(0,0.2]	C2, A1, C1, B1, and D3

## 3 Methods

### 3.1 XGBoost principle

XGBoost is a prominent and highly efficient boosting ensemble learning algorithm, representing an advanced evolution of the Gradient Boosted Decision Tree (GBDT) model (Yao et al., 2022; Xiong et al., 2024; Utkarsh, 2024). The predictive output of the XGBoost model is formulated as shown in Equation 4:


(4) $$\begin{array}{l} {\hat{y}}_{i}=\sum_{k=1}^{K}f_{k}\left(x_{i}\right),f_{k}\in F, \end{array}$$

where ŷ_*i*_ denotes the predicted value for the *i*-th sample; *K* represents the number of trees; *F* signifies the function space of the tree; *x*_*i*_ is the feature vector of the *i*-th data point; and *f*_*k*_ refers to the function learned by the *k*-th tree, which is characterized by its structure *q* and leaf weights *w*.

The loss function of the XGBoost model comprises two components, as shown in Equation (5):


(5) $$\begin{array}{l} L=\sum_{i=1}^{n}l\left(y_{i},{\hat{y}}_{i}\right)+\sum_{k=1}^{K}\Omega \left(f_{k}\right), \end{array}$$

where the first term represents the training error between the predicted value ŷ_*i*_ and the true target value *y*_*i*_; the second term denotes the sum of tree complexities, which serves as a regularization term to control the model's complexity, as presented in Equation 6:


(6) $$\begin{array}{l} \Omega \left(f\right)=\gamma T+\frac{1}{2}\lambda ∥w{∥}^{2}, \end{array}$$

where γ and λ are the penalty coefficients.

During the minimization process of the objective function defined in Equation 5, the incremental function *f*_*t*_(*x*_*i*_) is added at each iteration to reduce the loss function. The objective function at the *t*-th iteration is presented in Equation 7:


(7) $$\begin{array}{r} L^{\left(t\right)}=\overset{n}{\underset{i=1}{\sum }}l\left(y_{i},{\hat{y}}_{i}\right)+\overset{K}{\underset{k=1}{\sum }}\Omega \left(f_{k}\right)\\ \begin{array}{c} = \end{array}\sum_{i=1}^{n}l\left(y_{i},{\hat{y}}_{i}^{t-1}+f_{t}\left(x_{i}\right)\right)+\Omega \left(f_{k}\right). \end{array}$$

For Equation 7, the The sample set is defined in each leaf of t objective function is approximated using a second-order Taylor expansion. The *j*-th tree as *I*_*j*_ = {*i*|*q*(*x*_*i*_ = *j*)}. Here, $g_{i}=\partial_{{\hat{y}}_{i}^{\left(t-1\right)}}l\left(y_{i},{\hat{y}}_{i}^{\left(t-1\right)}\right)$ and $h_{i}=\partial_{{\hat{y}}_{i}^{\left(t-1\right)}}^{2}l\left(y_{i},{\hat{y}}_{i}^{\left(t-1\right)}\right)$ represent the first and second derivatives of the loss function, respectively. From these definitions, Equation 8 can be derived as follows:


(8) $$\begin{array}{r} L^{\left(t\right)}=\sum_{i=1}^{n}\left[g_{i}f_{t}(x_{i})+\frac{1}{2}h_{i}f_{t}^{2}(x_{i})\right]+\Omega (f_{t})\\ ≅\sum_{i=1}^{n}[g_{i}f_{t}(x_{i})+\frac{1}{2}h_{i}f_{t}^{2}(x_{i})]+\gamma T+\frac{1}{2}\lambda \sum_{j=1}^{T}w_{j}^{2}\\ ≅\sum_{j=1}^{T}[(\sum_{i\in I_{j}}g_{i})w_{j}+\frac{1}{2}(\sum_{i\in I_{j}}h_{i}+\lambda )w_{j}^{2}]+\gamma T \end{array}$$

Defining $G_{j}=\underset{i\in I_{j}}{\sum }g_{i}$ and $H_{j}=\underset{i\in I_{j}}{\sum }h_{i}$ leads to Equation 9:


(9) $$\begin{array}{l} L^{\left(t\right)}≅\sum_{j=1}^{T}\left[G_{j}w_{j}+\frac{1}{2}\left(H_{j}+\lambda \right)w_{j}^{2}\right]+\gamma T. \end{array}$$

The partial derivative with respect to *w*_*j*_ yields Equation 10:


(10) $$\begin{array}{l} w_{j}=-\frac{G_{j}}{H_{j}+\lambda }. \end{array}$$

Substituting the weights into the objective function yields Equation 11:


(11) $$\begin{array}{l} L^{\left(t\right)}≅-\frac{1}{2}\sum_{j=1}^{T}\frac{G_{j}^{2}}{H_{j}+\lambda }+\gamma T. \end{array}$$

A smaller loss function signifies enhanced model performance. A greedy algorithm is used to partition the subtree by enumerating feasible split points: a new split is added to existing leaves at each step, and the maximum gain is computed accordingly. The gain is then calculated as shown in Equation 12.


(12) $$\begin{array}{l} L_{Gain}≅\frac{1}{2}\left[\frac{G_{L}^{2}}{H_{L}+\lambda }+\frac{G_{R}^{2}}{H_{R}+\lambda }-\frac{G_{L}+G_{R}}{H_{L}+H_{R}+\lambda }\right]-\gamma , \end{array}$$

where the first and second terms represent the gains achieved by splitting the left and right subtrees, respectively, whereas the third term corresponds to the gain obtained without any split.

### 3.2 Attention mechanism

**Figure 4:**
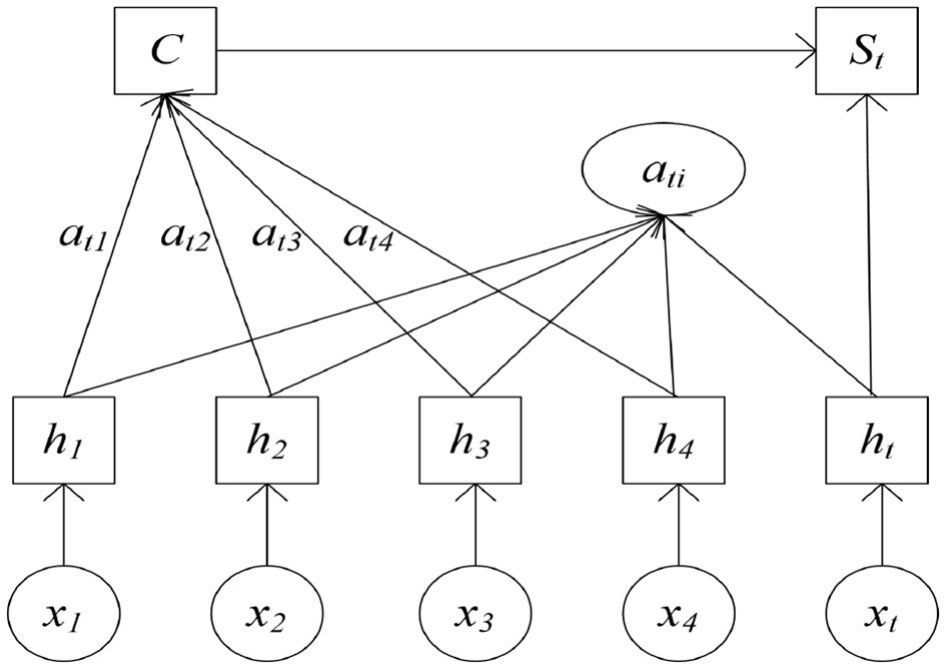
Structure of the attention cell.

The attention mechanism receives the output from the Stacking model as its input and adaptively assigns weights to its input features, thereby emphasizing the most relevant ones and suppressing less important features, consequently facilitating more accurate feature selection (Zhu et al., 2021; Wankhade et al., 2023). The structure of the attention mechanism is shown in Figure 4.

In Figure 4, *x*_1_, *x*_2_, …, *x*_*t*_ represents the inputs from multi-source fusion data; *h*_1_, *h*_2_, …, *h*_*t*_ corresponds to the output state values generated by the ensemble model, while *a*_*ti*_ signifies the adaptive attention weight assigned to each output; *s*_*t*_ denotes the final output.

By calculating the correlation between *h*_1_, *h*_2_, ⋯ , *h*_*t*_ and the current decoding time, the *e*_*t, i*_ for each influencing factor is obtained. These updated values are presented in Equation 13.


(13) $$\begin{array}{l} e_{t,i}=V^{T*}\tan h\left(Wh_{t}+Uh_{i}\right),i=1,2,\ldots ,t-1. \end{array}$$

According to the computed probability *e*_*t, i*_ of each influencing factor within the population, this value is then used to compute the attention weight for each output of the ensemble model. The updated output is presented in Equation 14.


(14) $$\begin{array}{l} a_{t,i}=\frac{\exp\left(e_{t,i}\right)}{\sum_{k=i}^{N_{t}}\exp\left(e_{k,i}\right)},i=1,2,\cdots \;,t-1. \end{array}$$

The hidden states *h*_1_, *h*_2_, ⋯ , *h*_*t*_ are weighted by their corresponding attention values and then linearly combined. The updated output is shown in Equation 15.


(15) $$\begin{array}{l} C=\sum_{i=1}^{N_{i}}a_{t,i}h_{i},i=1,2,\cdots \;,t-1 \end{array}$$

*S*_*t*_ represents the final output derived through the attention mechanism, as shown in Equation 16.


(16) $$\begin{array}{l} s_{t}=f\left(C,h_{t}\right), \end{array}$$

where *V, W, and U* represent the trainable parameters, which are continuously updated during model training.

### 3.3 Multi-model fusion for coal and gas outburst prediction

The performance of the Stacking model is directly influenced by the number of base models used. Using too few base models may not provide adequate diversity to effectively support the meta-model, while using too many could result in redundancy, higher computational costs, and a more intricate tuning process. Typically, 3–5 base models are recommended (Kumar et al., 2024; Zhao et al., 2024b).

**Figure 5:**
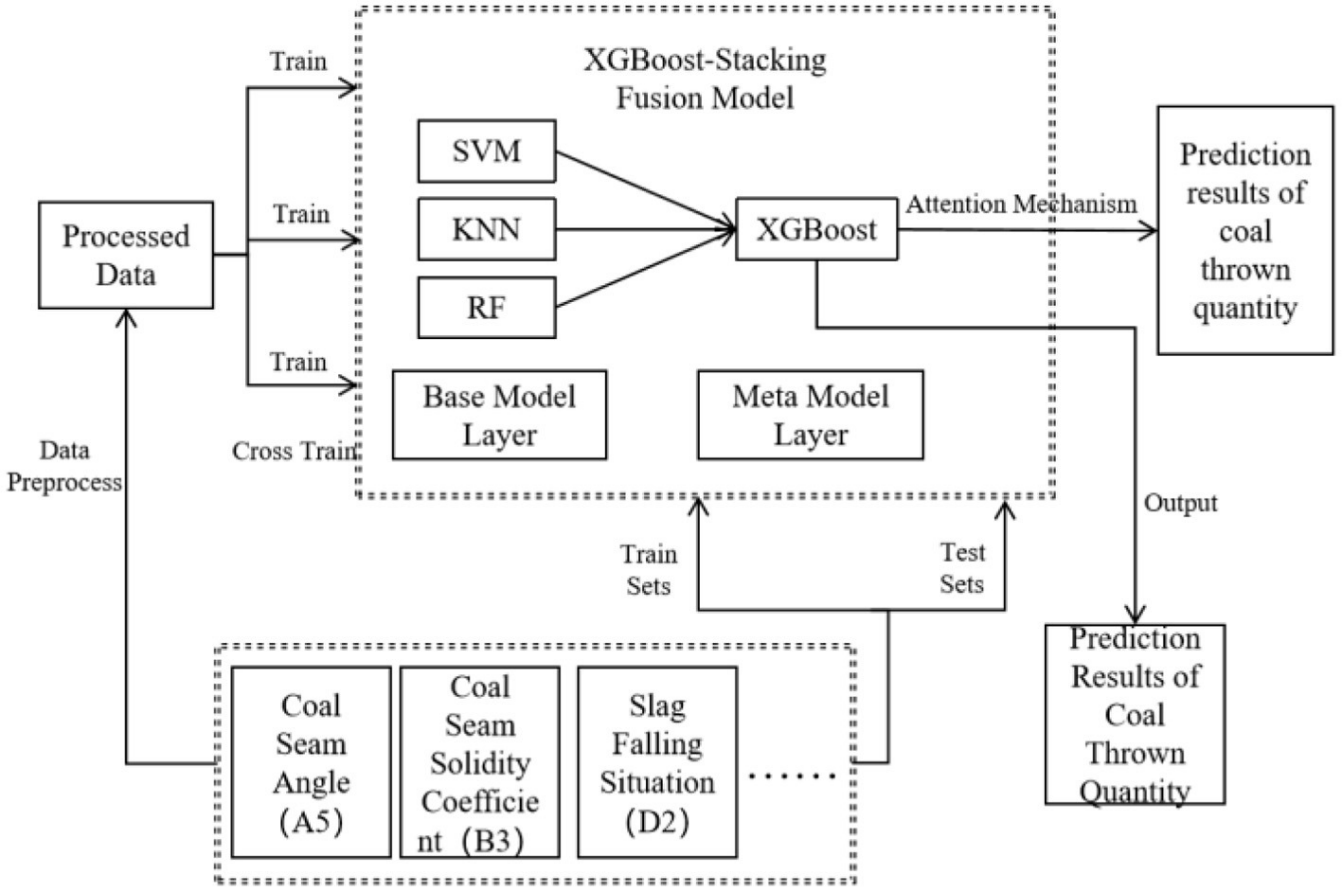
Prediction framework based on AXGBoost and Stacking.

Based on the predictive capabilities of various base learners, this article selected high-performing models as the first-layer base learners in the Stacking model. This selection is driven by the fact that base models with strong learning abilities contribute to improving the overall predictive accuracy of the ensemble. Specifically, RF, which uses the bagging technique, is preferred for its robust learning capacity and well-established theoretical foundation, making it applicable across a wide range of domains. SVM is selected for its unique strengths in handling small datasets, non-linear relationships, and high-dimensional regression problems. KNN is included due to its solid theoretical background and efficient training process, delivering strong practical performance. For the second layer, models with robust generalization capabilities are chosen to aggregate and correct biases from the multiple base learners in the training set while mitigating overfitting through ensemble strategies. Consequently, the Stacking ensemble model incorporates RF, KNN, and SVM as the first-layer base learners, with Attention-XGBoost serving as the meta-learner in the second layer. The overall architecture is shown in Figure 5.

**Figure 6:**
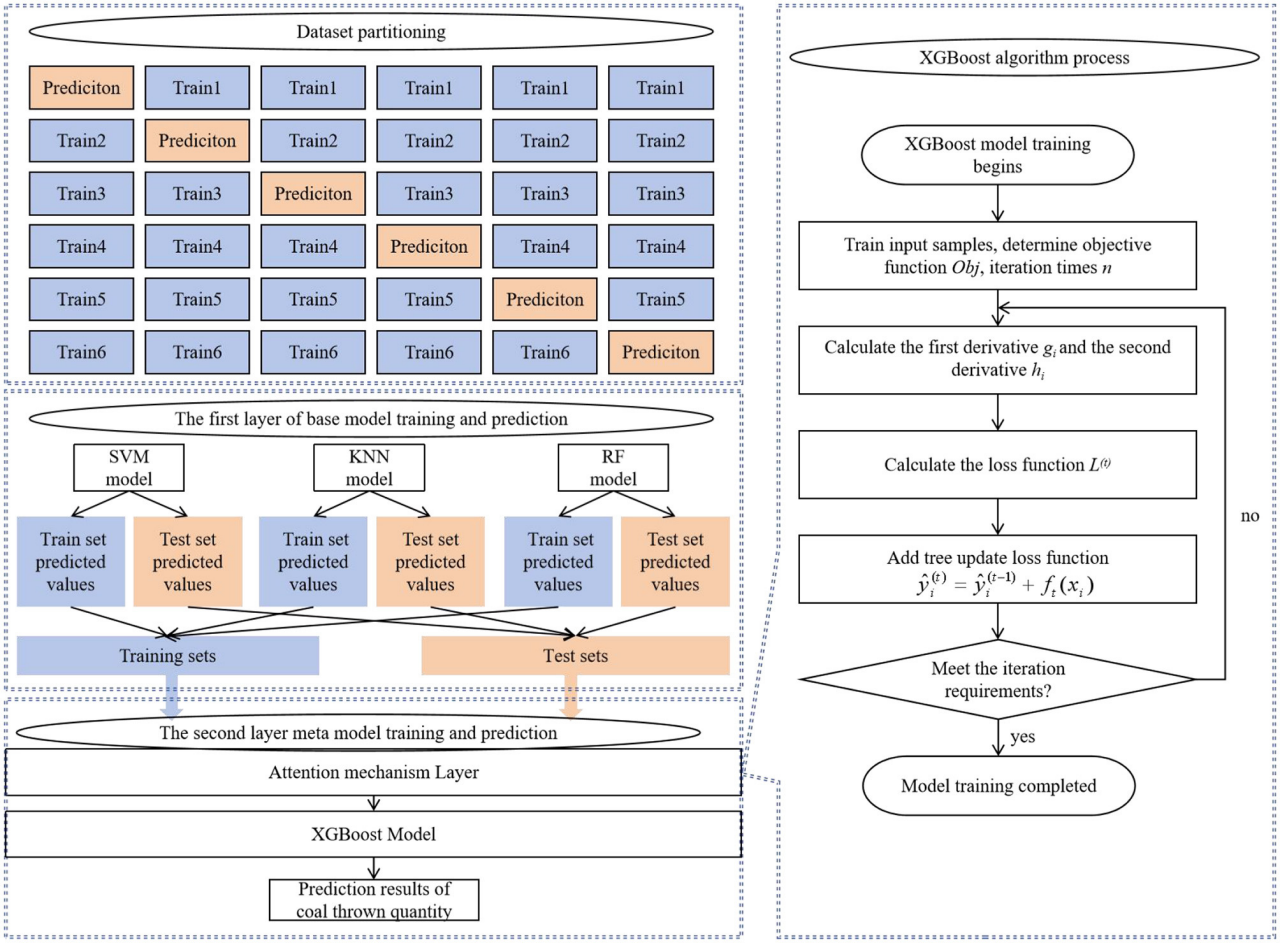
Coal thrown quantity prediction method based on multi-model fusion within a Stacking framework.

It is important to note that the training set for the meta-learner is derived from the outputs of the base learners. Directly utilizing the base learners' training data to form the meta-learner's training set could result in significant overfitting. To prevent the data from being redundantly learned by both layers and to avoid overfitting, an appropriate data usage strategy must be implemented. The dataset is first split into training and testing sets using cross-validation, with the three base learners making independent predictions. For each base learner, the original training dataset is partitioned into six mutually exclusive subsets, ensuring that no data IDs are repeated across subsets. For each base learner, one data subset is reserved as the validation set, while the remaining five subsets serve as the training set. Each base learner produces prediction results on its own validation subset. These predictions from the three base learners are then combined to form a new dataset, equal in size to the original dataset, as illustrated in Figure 6. This approach facilitates a comprehensive feature transformation from the original input features to the meta-learner's input features. Since each base learner's predicted data subset was excluded from its own training, this method guarantees that every data point is used only once during training, effectively preventing overfitting.

The training and prediction process of the AXGBoost-Stacking model is shown in Figure 6, and the detailed training procedure is outlined as follows:

Step 1: The coal and gas outburst dataset is defined as presented in Equation 17.


(17) $$\begin{array}{l} s=\left\{\left(y_{n},x_{n}\right),n=1,2,\cdots ,N\right\}, \end{array}$$

where *x*_*n*_ represents the feature vector of the *n*-th sample, *y*_*n*_ is the corresponding target (predicted) value, and *p* is the number of features, meaning that each feature vector can be expressed as (*x*_1_, *x*_2_,..., *x*_*p*_). Next, the dataset is partitioned into *Z* equally sized subsets: *S*_1_, *S*_2_,..., *S*_*z*_. The cross-validation between datasets as presented in Equation 18.


(18) $$\begin{array}{l} {\bar{S}}_{z}=S-S_{z}, \end{array}$$

where *S*_*z*_ denotes the *z*-th test set and ${\overset{¯}{S}}_{z}$ represents the corresponding training set.

Step 2: The training set ${\bar{S}}_{z}$ is fed into the first layer of the XGBoost-Stacking ensemble model, where three base learners are trained to obtain the base model *L*. Simultaneously, each sample *x*_*n*_ in the cross-validation test set *S*_*z*_ is passed through the trained base model *L* to generate the corresponding predictions.

Step 3: The output predictions from the three base learners are concatenated to form a new data sample, which is then used as the input for the second layer of the Stacking model. At this stage, a prediction algorithm that integrates the attention mechanism with XGBoost is used to aggregate these outputs and finalize the prediction of coal ejection volume.

In this study, the AXGBoost-Stacking model is implemented using the scikit-learn library in Python. A detailed description of the algorithm is provided in Table 4.

**Table 4:** AXGBoost-Stacking algorithm description.

**Algorithm: AXGBoost-Stacking model**
1. Def attention_3d_block 2. Initialize AXGBoost-Stacking model 3. Get input data train_X from DataSet 4. Get output(label) data train_Y from DataSet 5. Train_X = train_X.reshape((train_X.shape[0],1,train_X.shape[1])) train_Y = train_Y.reshape((train_Y.shape[0],1,train_Y.shape[1])) inputs: Input(train_X.shape[1], train_X.shape[2]) 6. Base_model_layer:Stacking_basemodel_output = Stacking(base_mode[model_1, model_2, model_3]) 7. attention.layer:attention_output = Flatten(attention_3d_block(Stacking_basemodel_output)) 8. meta_model_layer:Stacking_metamodel_output = Stacking(meta_mode[model]) 9. train iterater begin 10. Calculate the mean square error of the loss function based on the predicted and actual values 11. Train iterater end

## 4 Experimentation and evaluation

### 4.1 Model evaluation indicators

The multi-model Stacking prediction framework proposed in this study adopts the AXGBoost-Stacking model, which uses SVM, RF, and KNN as base learners and an attention-enhanced XGBoost model as the meta-learner. The Stacking ensemble learning algorithm enables a two-layer fusion of the SVM, RF, KNN, and XGBoost models. In addition to AXGBoost-Stacking model, three alternative Stacking models can also be constructed for comparative analysis:

1) The SVM-Stacking model uses RF, KNN, and AXGBoost as base learners, with SVM serving as the meta-learner.
2) The RF-Stacking model uses SVM, KNN, and AXGBoost as the base learners, with RF acting as the meta-learner.
3) The KNN-Stacking model uses SVM, RF, and AXGBoost as the base learners, with KNN acting as the meta-learner.

To evaluate the predictive performance of the AXGBoost-Stacking model and compare it with the individual predictive capabilities of the other three Stacking models, this study uses mean squared error (MSE), mean error (ME), and the F1-score [as defined in reference Xie et al. (2018)] as evaluation metrics. The formulas for calculating MSE and ME are provided in Equations 19, 20.


(19) $$\begin{array}{l} MSE=\frac{1}{n}\sum_{i=1}^{n}{\left(y_{i}-{\hat{y}}_{i}\right)}^{2} \end{array}$$

(20) $$\begin{array}{l} ME=\frac{1}{n}\sum_{i=1}^{n}|y_{i}-{\hat{y}}_{i}|, \end{array}$$

where *y*_*i*_ is true data, and ŷ_*i*_ is prediction data.

The calculation formula for the *F1*-score is presented in Equation 21.


(21) $$\begin{array}{l} F1\;=\;2\times \frac{Precision\times Recall}{Precision+Recall}, \end{array}$$

where *Precision* indicates the model's accuracy, and *R*_*ecall*_ represents the model's recall rate.

### 4.2 Comparison of prediction results

#### 4.2.1 Input feature contribution analysis

**Figure 7:**
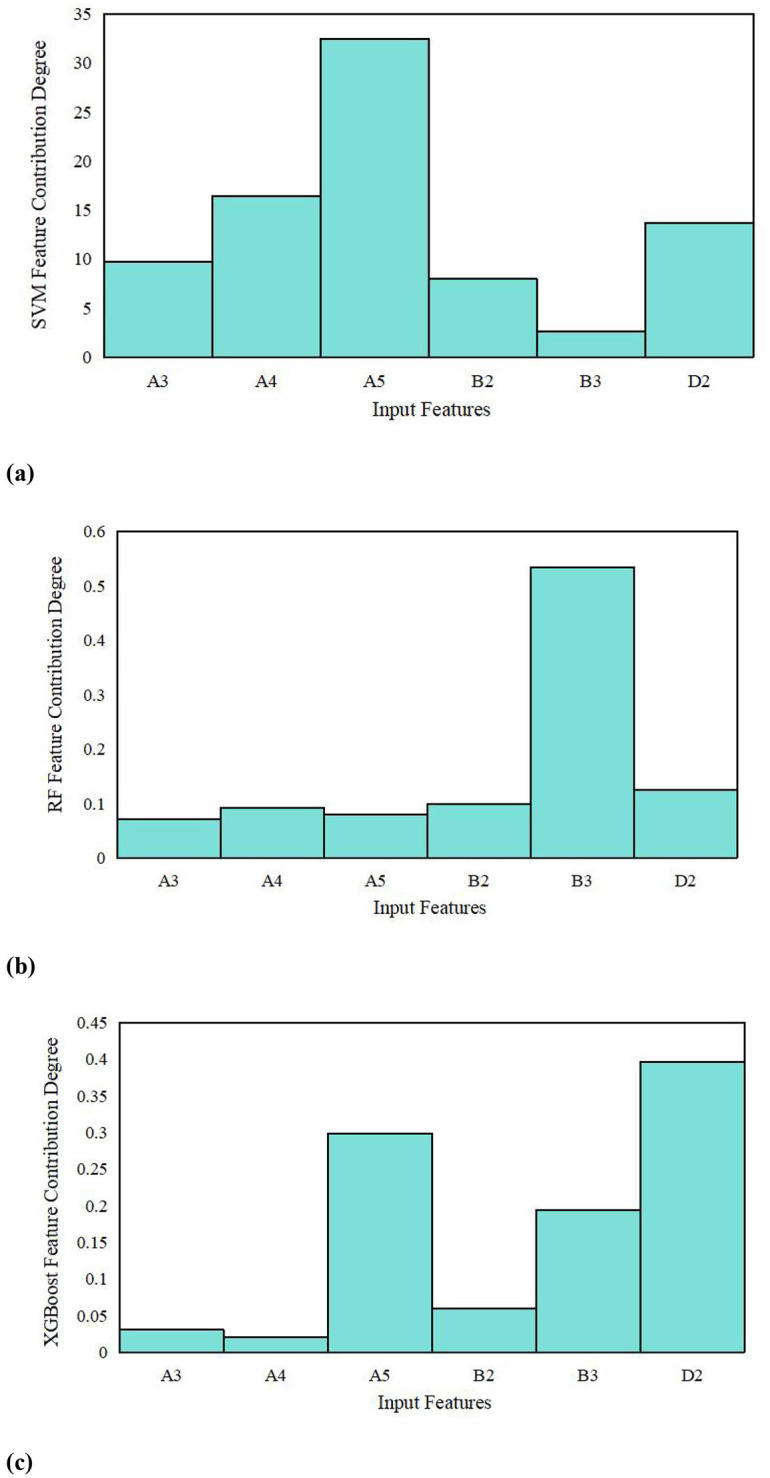
Contribution of input features to the prediction model. **(A)** SVM model input feature contribution. **(B)** RF model input feature contribution. **(C)** XGBoost model input feature contribution.

**Figure 8:**
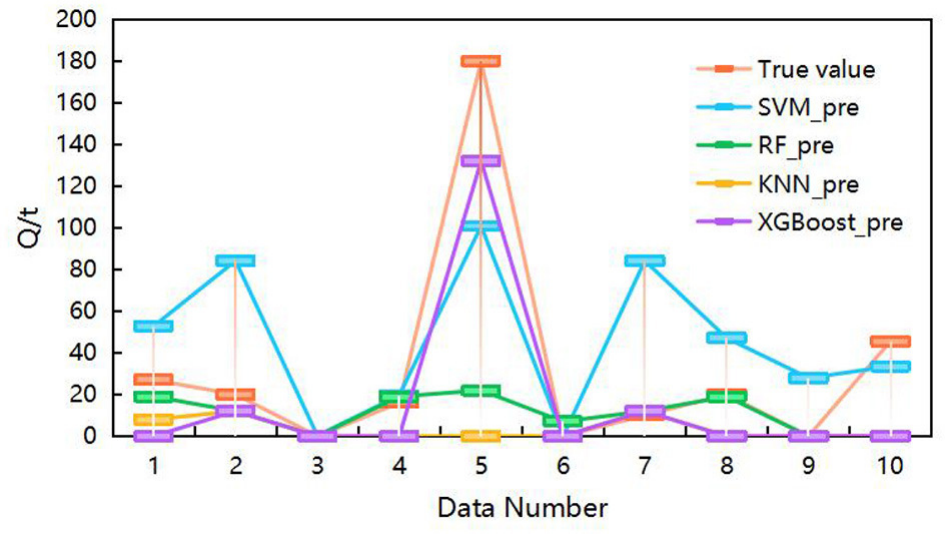
Comparison of the prediction performance of individual models.

As previously mentioned, this study uses the following features as model inputs: A5, B3, D2, A4, B2, and A3. The model's output is the coal thrown quantity, corresponding to the classification levels outlined in Table 2. Figure 7 illustrates the contribution analysis of input features for the SVM, RF, and XGBoost models. Additionally, the comparison of prediction performance among single models is provided in Figure 8.

As shown in Figure 7, A5, B3, and D2 exhibit high feature importance across different models. This finding is consistent with the Pearson correlation results presented in Table 3, indirectly validating that A5, B3, and D2 exert a greater influence on the model's predictive performance than other factors.

Based on the AUC values used for parameter tuning of the SVM, RF, KNN, and XGBoost models, the optimal parameter settings are listed in Table 5. As shown in Table 5, the prediction performance of the individual models, assessed by MSE and ME, is also compared. Combined with the results in Figure 8, it is clear that the XGBoost and SVM models exhibit superior predictive performance.

**Table 5:** Parameters of each model.

**Model**	**Model parameter**	**MSE**	**ME**	**F1**
SVM	Kernel = “linear”, *C* = 4	1,821.56	13.16	0.875
RF	n_estimators = 100, random_state = 0, n_jobs = −1	2,722.525	20.85	0.857
KNN	n_neighbors = 3	3,555.525	28.65	0.6
XGBoost	n_neighbors = 6, learining_rate = 0.09	582.725	16.25	0.6

#### 4.2.2 Performance analysis of Stacking model prediction

**Figure 9:**
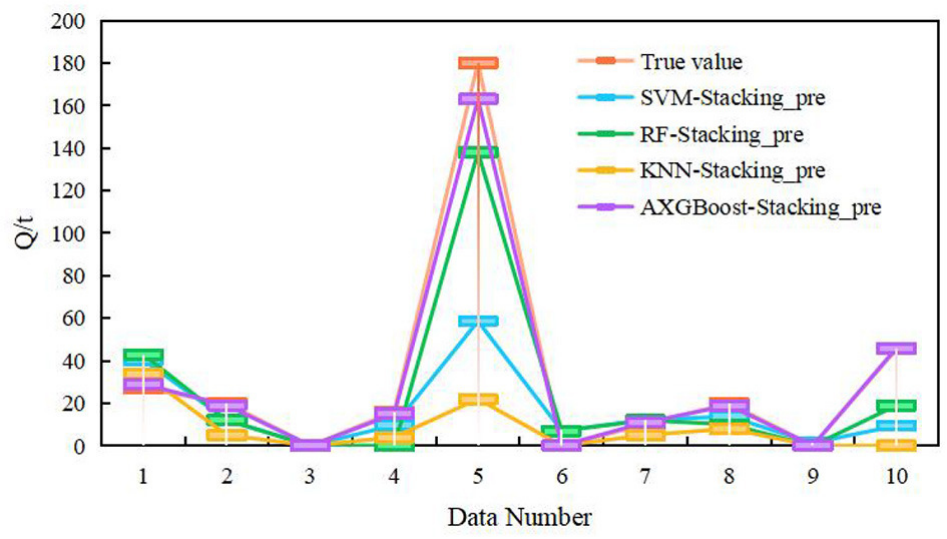
Comparison of the prediction performance of the Stacking model.

To evaluate the predictive performance of the Stacking ensemble model, SVM, RF, KNN, and XGBoost were used as meta-learners for comparative analysis. The selected parameters for SVM, RF, and KNN are consistent with those listed in Table 6. The resulting prediction results are shown in Figure 9, Tables 6, 7. The results highlight that the selection of base learners significantly impacts the final predictive performance.

**Table 6:** Parameters of each model.

**Serial number**	**True value**	**Level**	**SVM-Stacking**		**RF-Stacking**		**KNN-Stacking**		**AXGBoost-Stacking**	
			**Prediction value**	**Level**	**Prediction value**	**Level**	**Prediction value**	**Level**	**Prediction value**	**Level**
1	27	II	40.4188	II	43	II	34	II	29	II
2	20	II	12.0889	II	12	II	5	II	19	II
3	0	I	0.095	I	0	I	0	I	0	I
4	16	II	9.6556	II	0	I	4	II	15	II
5	180	IV	58.6095	IV	138	IV	22	II	163	IV
6	0	I	7.0851	II	7	II	0	I	0	I
7	10	II	12.0889	II	12	II	5	II	11	II
8	20	II	13.8117	II	10	II	8	II	19	II
9	0	I	1.3268	II	0	I	0	I	0	I
10	45.5	II	9.4444	II	19	II	0	I	46	II

**Table 7:** Parameters of each model.

**Model**	**MSE**	**ME**	**F1**
SVM-Stacking	1,641.318	15.38	0.823
RF-Stacking	319.525	7.75	0.857
KNN-Stacking	2,762.125	24.05	0.923
AXGBoost-Stacking	29.725	1.65	0.98

As shown in Tables 6, 7, the method proposed in this study achieves high prediction accuracy. Moreover, a comparison between Tables 5, 7 reveals that the Stacking model outperforms the individual models in terms of prediction accuracy. Compared with the prediction results reported in Xie et al. (2018), the approach utilized in this study demonstrates superior predictive performance.

## 5 Conclusion

This study incorporates advanced algorithmic techniques from the fields of AI and ML. In contrast to previous studies, particularly Xie et al. (2018), this study, within the Stacking ensemble framework, leverages multiple algorithms to interpret the data space and structure from diverse perspectives, enabling complementary strengths among models and yielding optimal prediction outcomes. Experimental results demonstrate that conducting feature contribution analysis before model construction effectively quantifies the importance of each feature. The Stacking ensemble learning algorithm exhibits strong predictive accuracy and holds significant application in coal and gas outburst prediction. The main contributions of this study are summarized as follows:

- Through Pearson's correlation analysis and feature importance evaluation, coal seam angle, coal seam solidity coefficient, and slag falling situation are identified as key factors contributing significantly to the prediction outcomes.
- Compared with individual models, the Stacking ensemble model effectively integrates the strengths of each base learner, thereby enhancing overall prediction accuracy.
- Due to the complexity of the model and the risk of overfitting caused by the small data size, cross-validation was adopted to prevent overfitting from occurring. In future research, adversarial learning or large-scale models will be introduced to effectively expand and validate the dataset.

## Data Availability

The raw data supporting the conclusions of this article will be made available by the authors, without undue reservation.
